# Transition of Women with Turner Syndrome from Pediatrics to Adult Health Care: Current Situation and Associated Problems

**DOI:** 10.3389/fped.2017.00028

**Published:** 2017-02-17

**Authors:** Hideya Sakakibara

**Affiliations:** ^1^Department of Gynecology, Yokohama City University, Medical Center, Yokohama, Japan

**Keywords:** Turner syndrome, transition, hormone replacement therapy, health-care surveys, adolescent

Turner syndrome (TS), a chronic childhood disease, requires growth hormone (GH) treatment for short stature in pediatrics and sex hormone replacement therapy (HRT) in the adult health-care setting with transition during adolescence managed by gynecologists or endocrinologists (1). However, many women with TS are reported to be receiving inadequate health care. Only 24 of 39 (63%) TS patients received regular follow-up in an Australian study (2), and 13 of 102 were lost to follow-up in a Belgian study (3). Thus, the current management of transition of TS patients seems to be inadequate. This is because there are limited numbers of gynecologists or endocrinologists who are familiar with adult TS, and a reliable management plan for TS during transition has not yet been fully established.

Turner syndrome women with complete or partial deletion of an X chromosome will develop various complications in each life stage (Figure 1). In addition to short stature, cardiovascular disease, kidney disease, and otitis media are often observed in childhood. GH therapy is performed for short stature in childhood and in adolescence they require HRT to acquire secondary sexual characteristics due to ovarian dysfunction. In adulthood, ovarian dysfunction causes infertility and osteoporosis. Endocrine/metabolic abnormalities, such as thyroid dysfunction, impaired glucose tolerance, hypertension, dyslipidemia, and gonadal tumors due to Y chromosome components are also observed. Therefore, all health problems that present during childhood should be followed-up in later life.

**Figure 1:**
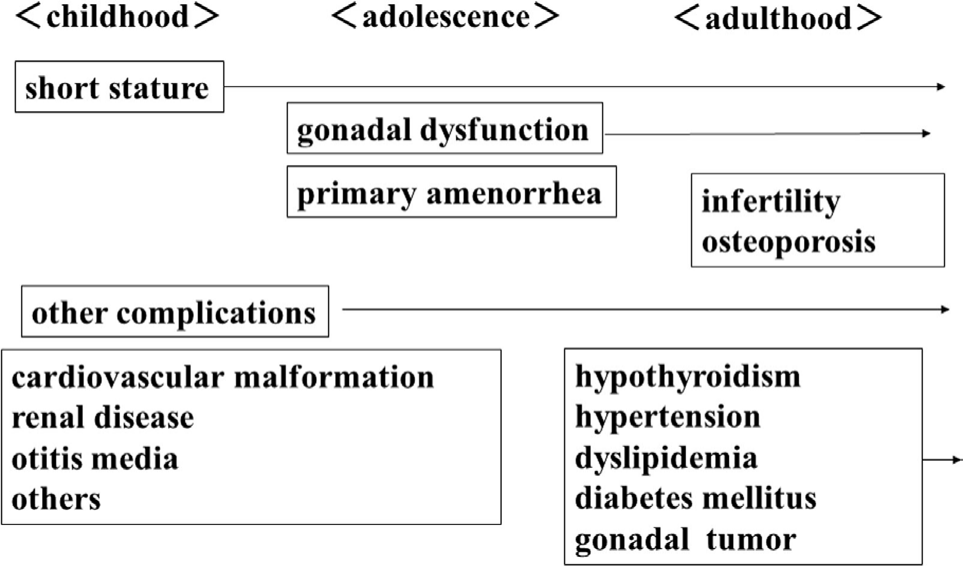
**Complications in women with TS**.

In our department, health management of TS adults is performed in Women’s Health Care Clinic by gynecologists who are familiar with the above conditions. Women’s health problems, due to the female-specific endocrine environment, change throughout every life stage (4). The salient points for TS health management are (1) commencement of HRT for ovarian dysfunction, (2) management of childhood onset complications, and (3) screening for complications that may develop in adulthood. At every visit, blood pressure and body weight were measured. Blood liver function, renal function, glucose tolerance, lipids, thyroid function, and urine analysis were measured once a year. Bone mineral density, ultrasound measurement of uterine size, and aortic MRI were performed at first visit. When a complication developed in a patient, she was referred to the relevant health-care professionals. Gawlik and Malecka-Tendera (5) have also demonstrated that all medical problems that present during childhood should be followed in adult life, with particular importance assigned to screening for hypertension, diabetes mellitus, dyslipidaemia, and osteoporosis.

It has been recommended in the guidelines (1) that transition care should be continued up to 18 years of age before transfer to adult health care, in which a gynecologist who is familiar with the unique problems of TS adults, such as ovarian dysfunction and infertility, is involved. HRT is recommended to start at 12–15 years old. However, in our department, the age at completion of GH therapy, in patients referred from pediatrics, was 17.7 ± 2.1 years old, the age of commencement of HRT was 17.9 ± 2.2 years of age, and the age at the time of transition was 23.4 ± 4.1 years of age. On the other hand, in survivors of childhood cancer, the starting age for HRT was 14.1 ± 1.2 years of age and the age of transition was 14.5 ± 1.9 years old (6). Our data shows that transition was not fully completed, although HRT was initiated and GH therapy had been completed. The transition from pediatrics was later than that recommended because of a delay in the end of GH therapy and initiation of HRT.

Since there is a lifetime increased risk of complications in TS women, the needs for a systematic and holistic approach in the provision of health care and the establishment of an optimal model of transition to adult health care in TS women are essential (7). A pediatric gynecology clinic was opened in Kanagawa Children’s Medical Center for the management of HRT in childhood and to ensure a smooth transition to our department from the time of GH therapy completion to starting HRT.

We conclude that in the future, gynecologists should be more involved in coordinating the initiation of HRT in order to create a smoother transition from pediatrics to adult health care for the management of women with TS.

## Author Contributions

The author confirms being the sole contributor of this work and approved it for publication.

## Conflict of Interest Statement

The author declares that the research was conducted in the absence of any commercial or financial relationships that could be construed as a potential conflict of interest.
